# Expression of Senescence Marker TIGIT Identifies Polyfunctional Donor-Reactive CD4+ T Cells Preferentially Lost After Kidney Transplantation

**DOI:** 10.3389/fimmu.2021.656846

**Published:** 2021-04-30

**Authors:** Amy C. J. van der List, Nicolle H. R. Litjens, Mariska Klepper, Michiel G. H. Betjes

**Affiliations:** Department of Internal Medicine, Section Nephrology and Transplantation, Erasmus MC, University Medical Center, Rotterdam, Netherlands

**Keywords:** alloreactive, T-cells, kidney transplantation, antigen-specific, senescence

## Abstract

Development of T-cell hyporesponsiveness to donor antigen may explain the substantial decreased risk for acute rejection in the years following kidney transplantation. The underlying mechanisms of donor-specific hyporesponsiveness (DSH) are largely unknown but may allow for lowering of immunosuppressive medication. Due to the onset of DSH being more rapid and pronounced in older recipients (+55 years), we hypothesized that immunosenescence/exhaustion of T lymphocytes would be a contributing factor. This study tested whether donor-reactive recipient T cells become hyporesponsive due to exhaustion from continuous stimulation by donor antigen. Circulating donor-reactive T cells of both young and elderly stable kidney transplant recipients (N=17) before and 3-5 years after transplantation were analyzed at the single cell level for expression of exhaustion markers by multi-parameter flow cytometry followed by unsupervised and unbiased clustering. Clusters containing cells of a particular expression profile with significant differential abundance after transplantation were identified and further analyzed. Unexpectedly, our results do not demonstrate an increase in exhausted donor antigen-reactive T cells post transplantation. Instead, we demonstrate a significant decrease in donor antigen-reactive CD4+ T cells expressing T cell immunoglobulin and ITIM domain (TIGIT) long after transplantation. Further analysis at earlier timepoints indicated that this decrease is already present at six months post transplantation. Characterization of these CD4+ T donor-reactive cells expressing TIGIT revealed them to have a predominantly central and effector memory T cell phenotype and a highly poly-functional cytokine expression profile. This study has therefore identified TIGIT as a marker for a previously undescribed polyfunctional donor-reactive CD4+ T cell population whose decline following kidney transplantation may explain development of DSH.

## Introduction

While the majority of acute T cell mediated rejections occur within the first 3-6 months after transplantation, the incidence declines to virtually zero by 3-5 years post kidney transplantation (KT) (1). This decreased risk is thought to arise from a gradual decrease in alloreactivity of T-cells to donor antigen while response to alloantigen of a third party is retained. A better understanding of the development of donor-specific hyporesponsiveness (DSH) in recipient T cells could guide lowering of immunosuppressive medication in the years after transplantation. Less immunosuppression would vastly reduce the risk of infection, cancer and cardiovascular disease, long-term unwanted side effects especially prevalent in elderly kidney transplant recipients (2, 3).

Past studies have looked into clonal deletion, anergy and active regulation by regulatory T cells as possible mechanisms contributing to DSH development, however, the question has not been fully resolved (4–9). In recent years, T-cell exhaustion has been recognized as leading to T cell hyporesponsiveness in the context of chronic infection and cancer (10). A similar mechanism may be playing a role in transplant recipients with continuous stimulation by donor antigen leading to non-functional T cells with an exhausted phenotype. The loss of function in exhausted T cells progresses gradually and depends on factors such as antigen load, the duration of antigen stimulation, loss of help from CD4+ T cells and increased expression of co-inhibitory receptors, including programmed cell death 1 (PD-1), T cell immunoreceptor with Ig and ITIM domains (TIGIT), and T cell immunoglobulin mucin 3 (TIM-3) (10, 11). Eventually, exhausted T cells will lose the ability to proliferate or produce interleukin (IL)-2 followed by a decreased ability to secrete effector molecules, including inflammatory cytokines and granzymes (11).

Previous studies looking into donor-reactive T cells have been hampered due to a lack of a donor-reactive T cell marker. These studies had to rely solely on T cell proliferation and cytokine-producing assays but could not detect non-proliferating, non-cytokine producing T cells. We have previously shown that donor-reactive T cells can be identified by expression of the co-stimulatory molecule CD137 after short-term alloantigen stimulation (12). This allows for identification and detailed characterization of alloreactive T cells at the single cell level.

In this study, we tested the hypothesis that donor-reactive recipient T cells become exhausted by continuous stimulation by donor antigen. Unbiased clustering was used to compare the expression of 7 exhaustion markers on CD137-expressing (donor antigen-specific) T cells prior to and 3-5 years after kidney transplantation in 17 stable KT recipients.

## Materials and Methods

### Study Design

In an ongoing study (Gandalf study, project number: 18PhD08, funded by the Dutch Kidney Foundation) the effects of age on donor-reactive T cells are evaluated by studying a cohort of young (≤45 years) and elderly (>54 years) kidney transplant recipients. From these cohorts, 17 recipients were selected (**Table 1**) of which heparinized peripheral blood samples were obtained prior to and at 3-5 years after kidney transplantation. Only recipients with stable graft function without signs of chronic rejection and/or previous T-cell depleting therapy were included. All recipients received a similar immunosuppressive regimen. This included a non-depleting induction therapy with basiliximab (Simulect^®^, Novartis) and maintenance therapy with tacrolimus and (Prograf^®^, Astellas Pharma), mycophenolate mofetil (MMF) (Cellcept^®^, Roche) (**Table 1**). This study was approved by the Medical Ethical Committee of the Erasmus MC and all participating kidney transplant recipients (MEC No. 2018-048) and healthy individuals (MEC No. 2018-1623) gave written informed consent to participate in this study. This study was conducted in accordance with the Declaration of Helsinki and the Declaration of Istanbul and in compliance with International Conference on Harmonization/Good Clinical Practice regulations.

**Table 1:** Study population characteristics.

Number of individuals	N=17
Age in years #,*	
* Young cohort (≤45)*	33 (19-45)
* Elderly cohort(>54)*	66 (57-77)
Male, n(%)	9 (55.6%)
Time after transplantation in years,	4(3-5)
Donor age in years #,*	56 (26 -74)
Donor Male, n(%)	6 (35%)
First kidney transplant, n(%)	17 (100%)
Transplantation from living donor, n(%)	16 (94%)
Number of mismatches HLA,*	
* HLA class I*	2 (1-4)
* HLA class II*	1 (0-2)
Renal replacement therapy, n(%)	
* No (pre-emptive)*	10 (59%)
* Hemodialysis*	4 (24%)
* Peritoneal dialysis*	3 (18%)
Underlying kidney disease, n(%)	
* Nephrosclerosis/arteriosclerosis/hypertension*	3 (18%)
* Primary glomerulopathy*	3 (17%)
* Diabetic nephropathy*	2 (12%)
* Polycystic kidney disease*	5 (29%)
* Other*	3 (18%)
* Unknown*	1 (6%)
Immunosuppressive regimen at post KT timepoint	
* Tacrolimus and MMF*	16 (94%)
* Tacrolimus and Everolimus*	1 (6%)

#, mean age (range); *, at time of transplantation.

### PBMC Isolation

Peripheral blood mononuclear cells (PBMCs) were isolated from heparinized peripheral blood samples on the day of blood sampling using Ficoll-Paque Plus (GE healthcare, Uppsala, Sweden) and stored at -150°C until further use as described previously (13).

### CD3+ T-Cell Depletion of Allogeneic Stimuli

Recipient PBMCs were stimulated with CD3-depleted donor cells. As controls, a portion of recipient PBMCs were left unstimulated and a portion were stimulated with CD3-depleted non-donor allogeneic stimulator cells (third party). Third party stimulator cells were selected for having an equal number (but different) HLA-mismatches with the tested recipient as the donor. Allogeneic donor and third party stimulator PBMCs were depleted of CD3+ T cells using CD3 microbeads according to manufacturer’s instruction (Miltenyi Biotec, Bergisch Gladbach, Germany). Efficiency of CD3 depletion was evaluated using flow cytometry to ensure allogeneic stimuli were >95% depleted of CD3+ T cells. Recipient PBMCs and CD3-depleted allogeneic stimulator cells were allowed to rest for at least 6 hours at 37°C before use.

### CD137 Multi-Parameter Flow Cytometric Assay

T cells of stable kidney transplant recipients prior to and at 3-5 years after transplantation were evaluated following stimulation of 3-5 million recipient PBMCs with CD3-depleted allogeneic stimulator cells at a 1:1 ratio for 18-24 hours. Stimulation was performed in polystyrene tubes (BD, Erembodegem, Belgium) in the presence of co-stimulation anti-CD49d (1 µg/mL; BD) as has been described in detail (12). In addition, frequencies and median fluorescence intensity (MFI) of TIGIT-expressing donor-reactive CD137+CD4+ T cells were determined in the first year after transplantation in a separate cohort. For this purpose, samples prior to and at month 6 and month 12 after kidney transplantation of 6 stable kidney transplant recipients per timepoint were included. After stimulation, cells were washed and cell surface stained using antibodies to characterize cell type and exhaustion markers (**Supplementary Table 1**). Briefly, staining included a 15-minute incubation with Fixable Viability Stain-780 (FVS780; BD) to exclude dead cells. Upon washing, the cells were stained for 30 minutes at room temperature in BD Horizon™ Brilliant Stain Buffer with antibodies directed to CD3, CD4, CD8 to identify T cells and inclusion of APC-H/Cy7-labeled antibodies directed to CD14, CD19 and CD56 to further exclude any unwanted cells (DUMP channel) from the analysis (**Supplementary Table 1**). Alloreactive T cells were identified using an antibody directed to CD137 (12). Antibodies directed to the following exhaustion markers were included: CD160, Lymphocyte-activation gene 3 (LAG3), PD-1, TIM-3, TIGIT, CD244, and cytotoxic T-lymphocyte-associated protein 4 (CTLA-4) (**Supplementary Table 1**). Upon another wash, stained samples were fixed with 1% formaldehyde (Scharlau, Sentmenat, Spain) and stored overnight at 4˚C to be measured the following morning on the LSR Fortessa cell analyzer (BD). We stored 0.5-1 million viable T cells for each measurement. Viable T cells were identified by gating lymphocytes using the forward and side scatter characteristics after which singlets were identified by forward scatter area and height. The BV510 channel was used to identify CD3-expressing T cells while excluding for cells which are positive for APC-Cy7 antibodies labeling dead cells, CD14 (monocytes), CD19 (B-cells), and CD56 (natural killer cells). Analysis of the expression of exhaustion markers was done in an unsupervised manner using Flow Self-Organizing Map (FlowSOM) and diffcyt R-based tools as described in more detail below. Traditional flow cytometric analysis was done using Kaluza software version 2.1 (Beckman Coulter, Woerden, Netherlands).

### FlowSOM Clustering

To determine expression profiles, data obtained by flow cytometry was analyzed using the dimensionality reduction technique, FlowSOM, a package freely available on Bioconductor to be used with the statistical programming language, R (14, 15). First, we created a FlowSet class object in R using FlowCore (Bioconducter package) which contained the complete expression profile of the gated CD3 cells of each sample. The FlowSet was compensated, logically transformed and then used to build a self-organizing map (SOM). Due to CD137-expressing cells making up less than 1% of the total population of CD3 T cells, we assigned the cells to 625 clusters to preserve the heterogeneity of our cells of interest. The SOM assigned each cell to 1 of these 625 clusters (a grid of 25 x 25) based on their complete expression profile of markers for cell type (i.e. CD4, CD8), exhaustion phenotype (CD160, LAG3, PD-1, TIM-3, TIGIT, CD244, CTLA-4) and CD137, a marker to identify alloreactive T cells. Unwanted cells (i.e. CD14+, CD19+, CD56+ and dead cells) were pooled in a DUMP-channel and clusters with high MFI for this channel were excluded from the analysis. Clusters were visualized with a minimal spanning tree (MST) in which each cluster is represented as a ‘node’ with the mean expression of each marker represented as a slice of a pie chart.

### Characterization of TIGIT-Expressing Donor-Reactive CD137+ CD4+ T Cells

The CD137 multi-parameter flow cytometric assay was used to determine phenotype and functional characteristics of CD137+ CD4+ TIGIT+ T cells. The phenotype was characterized in 3 healthy individuals using the CD137 multi-parameter flow cytometric assay described above. The antibodies included to characterize the phenotype of TIGIT-expressing CD137+CD4+ T cells were directed to CCR7, CD45RA, CD28, and CD27 (**Supplementary Table 1**). Function was characterized for 13 PBMC samples through comparing frequencies of cytokine producing T cells between TIGIT+ and TIGIT- CD137+CD4+ T cells upon stimulation of PBMCs as described in detail before (12). Briefly, PBMCs were left unstimulated, were stimulated with CD3-depleted cells or, as a positive control, a combination of phorbol myristate acetate (PMA) (50 ng/ml; Sigma Aldrich, St Louis, MO, USA) and ionomycin (1 μg/ml; Sigma Aldrich) in presence of a protein transport inhibitor (Ebioscience, Life technologies, Bleijswijk, The Netherlands) for 15 hours. Intracellular cell staining was performed upon permeabilization using antibodies directed to IL-2, interferon gamma (IFN)-γ, tumor necrosis factor (TNF)-α and CD137 after cell surface staining for CD3, CD4 and TIGIT (**Supplementary Table 1**).

### Statistical Analyses

Data are presented as median (range) or proportions of the study population and the non-parametric paired Wilcoxon matched pair test was used to compare frequencies and median fluorescence intensity (MFI) of TIGIT-expressing CD4+CD137+ T cells prior to and at different timepoints post transplantation. The statistical method, diffcyt, was used to test for significant differences in cell abundance within each node, specifically comparing abundance between pre- and post-transplant timepoints of each sample (16). This differential analysis is conducted using the edgeR package to calculate empirical Bayes moderated tests at the cluster level (17, 18). First, a contrast matrix was created wherein each row indicated a sample and a column specified the comparison of interest for the differential test (i.e. the combination of model coefficients that is assumed to equal zero under the null hypothesis). The FlowSOM output, namely cluster cell counts and cluster medians (median marker expression for each cluster and sample), was used as input for diffcyt. EdgeR normalized for the total number of cells per sample and statistical power was increased by sharing information on variability (i.e. variance across samples for a single cluster) between clusters. Differential test results were returned in the form of p-values and adjusted p-values by the edgeR moderated test. Adjusted p-values were calculated using the “Benjamini Hochberg” procedure with the false discovery rate set to 0.1 or 10%. The clusters with an adjusted p-value below 0.05 were termed “top differential abundance” or topDA clusters. The difference in abundance of cells originating from pre or post timepoints within the topDA clusters was calculated as a log fold change (logFC) using the formula:

$$\text{log}\text{FC}=\text{log}2\left(\frac{\text{cell}\;\text{count}\;\text{post}\;\text{Tx}}{\text{cell}\;\text{count}\;\text{pre}\;\text{Tx}}\right)$$

The adjusted p-value and logFC per cluster were visualized using a volcano plot. In order to center the volcano plot at 0 on the x-axis the mean logFC for each sample was subtracted from the logFC value of each cluster calculated for that sample. The complete expression profile of each topDA cluster was visualized using a heatmap with MFI per marker calculated over all cells within a cluster.

## Results

### Baseline Characteristics of the Study Population

Clinical and demographic characteristics of the 17 stable KT recipients included in this study are shown in **Table 1**. The median age (range) at transplantation of the young and elderly group was 33 (19-45) years and 66 (57-77) years, respectively. Post-transplantation T cell analysis was done at a median of 4 (range 3-5) years.

### Unsupervised Multi-Parameter Analysis Identifies a Significant Decline in TIGIT-Expressing CD4+CD137high T Cell Clusters Post Transplantation

**Figure 1:**
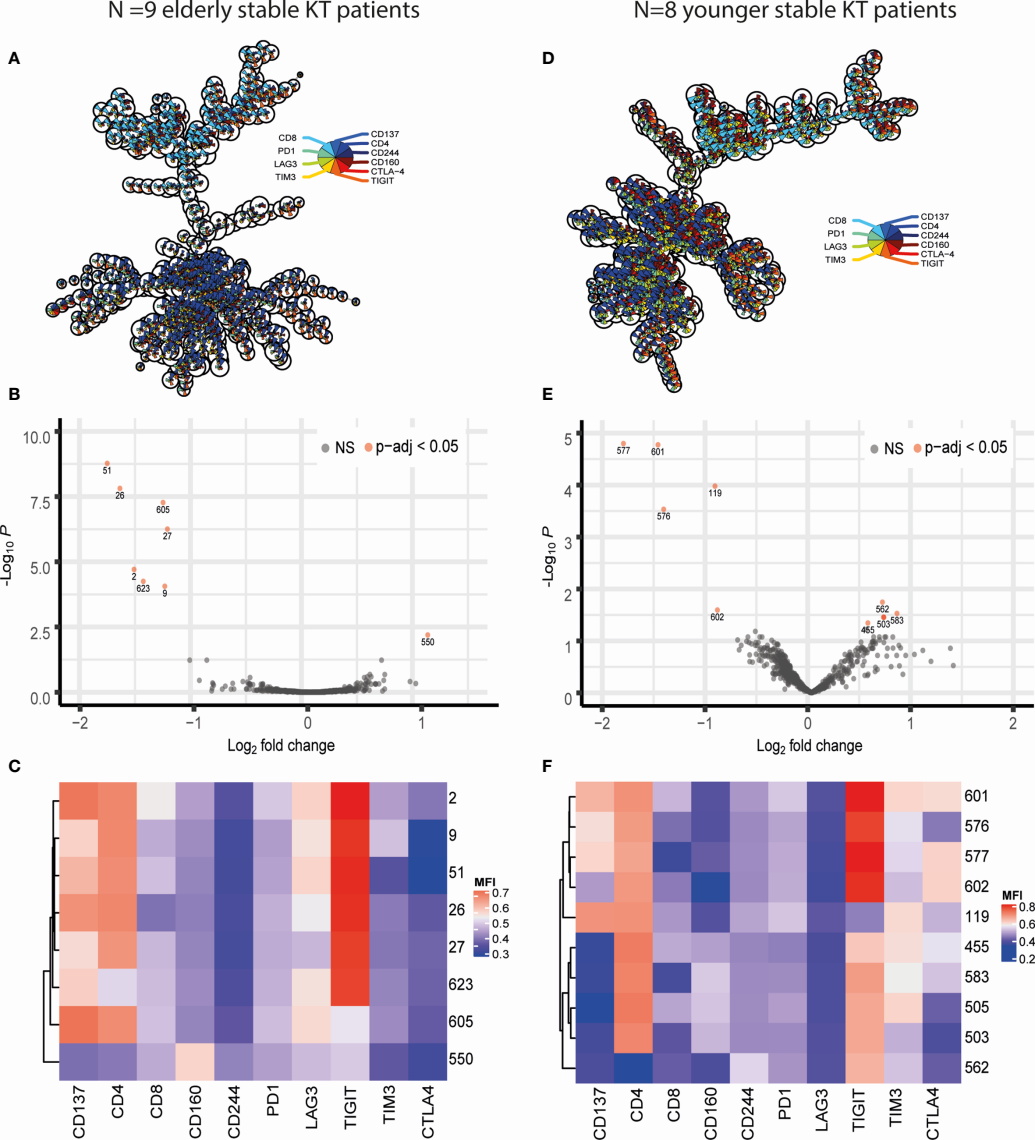
Visualization of FlowSOM and diffcyt results obtained through comparing CD3 T cells stimulated with donor antigen from pre and post KT. Panels on the left **(A–C)** contain data obtained from comparing pre- vs post-KT donor-stimulated T cells in N=9 elderly recipients while those on the right **(D–F)** resulted from comparing pre- *vs* post-KT donor-stimulated T cells in N=8 young KT recipients. **(A, D)** Minimal spanning trees (MST) represent the 625 clusters created with FlowSOM. Each ‘node’ representing a cluster contains a pie chart which illustrates the mean marker values. The nodes placed near each other have similar marker expression profiles. **(B, E)** Volcano plots of diffcyt results for each FlowSOM cluster showcasing the negative log base 10 of the adjusted p-values on the y axis and log base 2 of the fold change from pre to post KT on the x axis. TopDA clusters with a p adjusted value below 0.05 (highly significant) are highlighted in red. **(E, F)** The median fluorescence intensity (MFI) expression profile of each topDA cluster is illustrated in a heatmap with rows representing each cluster and columns the markers of interest. ns, non-significant.

Differential expression of exhaustion markers on T cells before and after KT was initially analyzed in the elderly recipients as the largest effects were expected to be present within this group. CD3+ T lymphocytes from all samples were arranged into 625 clusters of a self-organizing map (FlowSOM) based on their expression profile. Of the 625 clusters, only 37 (5.92%) classified as ‘DUMP’ or unwanted cells based on co-expression of cell death or non-T cell markers (**Figure 1A**). There was a clear distinction between CD4+ and CD8+ T cells with the majority of clusters, 442 (70.72%), representing CD4+ T cells and 142 (22.72%) comprising of CD8+ T cells (**Figure 1A**). Twenty-five clusters (4%) were made up of donor-reactive T cells due to their high expression of CD137. The majority of these 25 CD137high clusters contained CD8+ (N = 16, 64%) or CD4+ (N = 8, 32%) T cells with 1 cluster being double negative for CD4 and CD8 (**Figure 1A**). Testing for differential abundance (DA) of cells within a cluster revealed that 8 clusters had a significant change (p-adjusted <0.05) in abundance post KT (**Figure 1B**). Of these 8 “top differential abundance” or topDA clusters, cluster 550 was excluded due to its classification as DUMP. The 7 relevant topDA clusters all showed a decrease in abundance post KT with a median log fold change (logFC) of -1.55 ranging from -1.81 to -1.29 (**Figure 1B**). Strikingly, the topDA clusters contained CD4+ T cells with a high expression of CD137 and all but one (cluster 605) had similar expression profiles with TIGIT being the only exhaustion marker highly expressed (**Figure 1C**). Also noteworthy is the low expression of LAG3 present in the majority of these clusters. Similar analyses were repeated on CD3+ T cells from the same KT recipients stimulated with antigen containing an equal number but different HLA-mismatches than those observed for the donor kidney (known as third party). Only three top DA clusters, all of which contained CD137high CD4+ T cells expressing TIGIT were identified and median (range) logFC per cluster was relatively low at -0.855 (-0.86 to -0.85) (**Supplementary Table 2, Supplementary Figure 1**).

### Age-Independent Decrease in Donor-Reactive CD137+ CD4+ T Cells Expressing TIGIT

A similar approach was taken for donor-reactive T cells of 8 KT recipients from a younger cohort resulting in 10 topDA clusters with a p-adjusted value below 0.05 (**Figures 1D, E**). The 4 topDA clusters with the most significant differential abundance contained CD4+CD137high T cells (**Figures 1E, F**). Similar to the elderly, all but one of these CD4+CD137high topDA clusters had a high expression of TIGIT and declined substantially in frequency post KT with a negative median logFC per cluster of -1.39 ranging from -1.85 to -0.92 (**Figure 1E**). Unlike the elderly KT recipient cohort, two of the CD4+CD137highTIGIThigh topDA clusters, clusters 601 and 577, had a weak co-expression of CTLA-4 (**Figure 1F**). In addition, CD4+CD137highTIGIThigh cluster 601 also expressed a low level of TIM3. Also unique to the younger cohort is the identification of 5 topDA clusters which have a significant increase in abundance of CD4+ T cells which lowly express TIGIT but are negative for CD137. The median (range) logFC of these CD4+CD137-TIGIThigh T cell topDA clusters is 0.69 (0.55-0.83) (**Figure 1E**). The control experiments with third party stimulation showed no significant changes in abundance of clusters over time, including alloreactive CD137+ T cell clusters (**Supplementary Table 3**). Overall, the results obtained in the younger group are in accordance with the elderly group and identify a decline in TIGIT-expressing donor-reactive (CD137high) CD4+ T cell clusters 3-5 years after transplantation.

### Unsupervised Multi-Parameter Analysis Verified by Traditional Flow Cytometry Analysis

**Figure 2:**
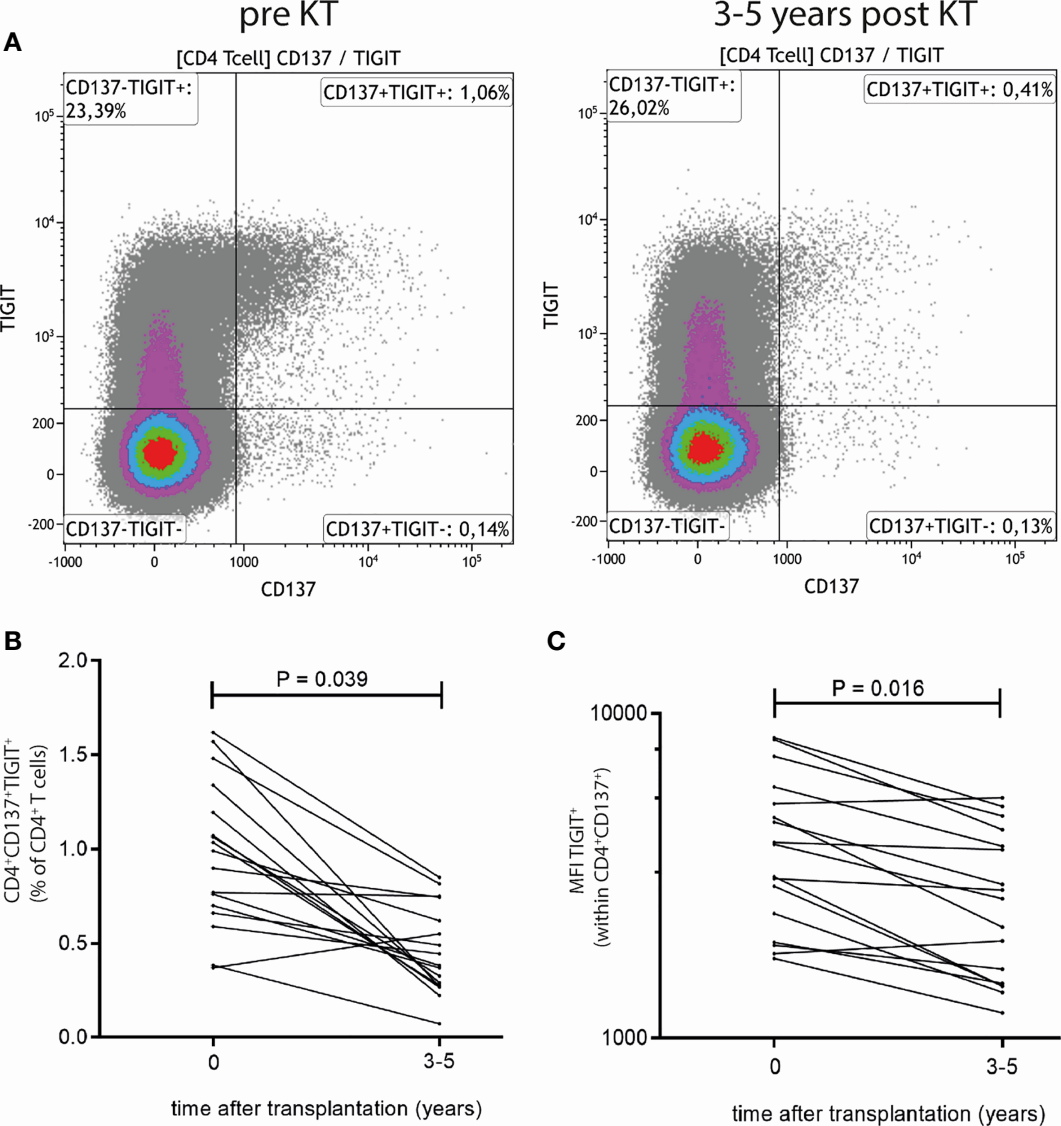
Traditional 2D flow cytometric analysis confirms reduction in frequency and MFI of TIGIT expression within donor-reactive CD4+CD137+ T cells. **(A)** Typical flow cytometric example of TIGIT expression prior to (left plot) and 3-5 years after KT (right plot). Percentages in upper right quadrant indicate CD4+ T cells which express CD137 and TIGIT. **(B)** Percentages of CD4+CD137+TIGIT+ as a percentage of total CD4+ T cells and **(C)** MFI of TIGIT expression within CD4+CD137+ T cells prior to and 3-5 years post KT for 9 elderly and 8 young KT recipients are depicted. The Wilcoxon signed rank test was used to compare samples prior to KT to those 3-5 years after KT and a P-value < 0.05 was considered statistically significant.

The decrease post KT in donor-reactive TIGIT-expressing CD4+CD137high T cells was verified by traditional 2D flow cytometric analysis (**Figure 2**). A representative flow cytometric example of percentages and median fluorescence intensity (MFI) of TIGIT-expressing CD4+CD137high T cells prior to and 3-5 years after KT is given in **Figure 2A**. A significant decrease in both the percentage of donor-reactive CD4+ T cells expressing TIGIT (P = 0.039) as well as the MFI of TIGIT (P = 0.016) was observed (**Figures 2B, C**). The median (range) percentage of CD4+ T cells dropped from 0.88% (0.37%-1.62%) of CD4+ T cells pre KT to 0.52% (0.27%-0.85%) post KT. Likewise, the median (range) MFI of TIGIT decreased from 5609 (3952-8429) prior to KT to 4146 (2197-5498) post KT. Unlike within CD4+ cells, neither the percentage of CD8+ T cells co-expressing CD137 and TIGIT nor the MFI of TIGIT within CD8+CD137+ T cells changes prior to vs 3-5 years post transplantation (**Supplementary Figure 2**).

**Figure 3:**
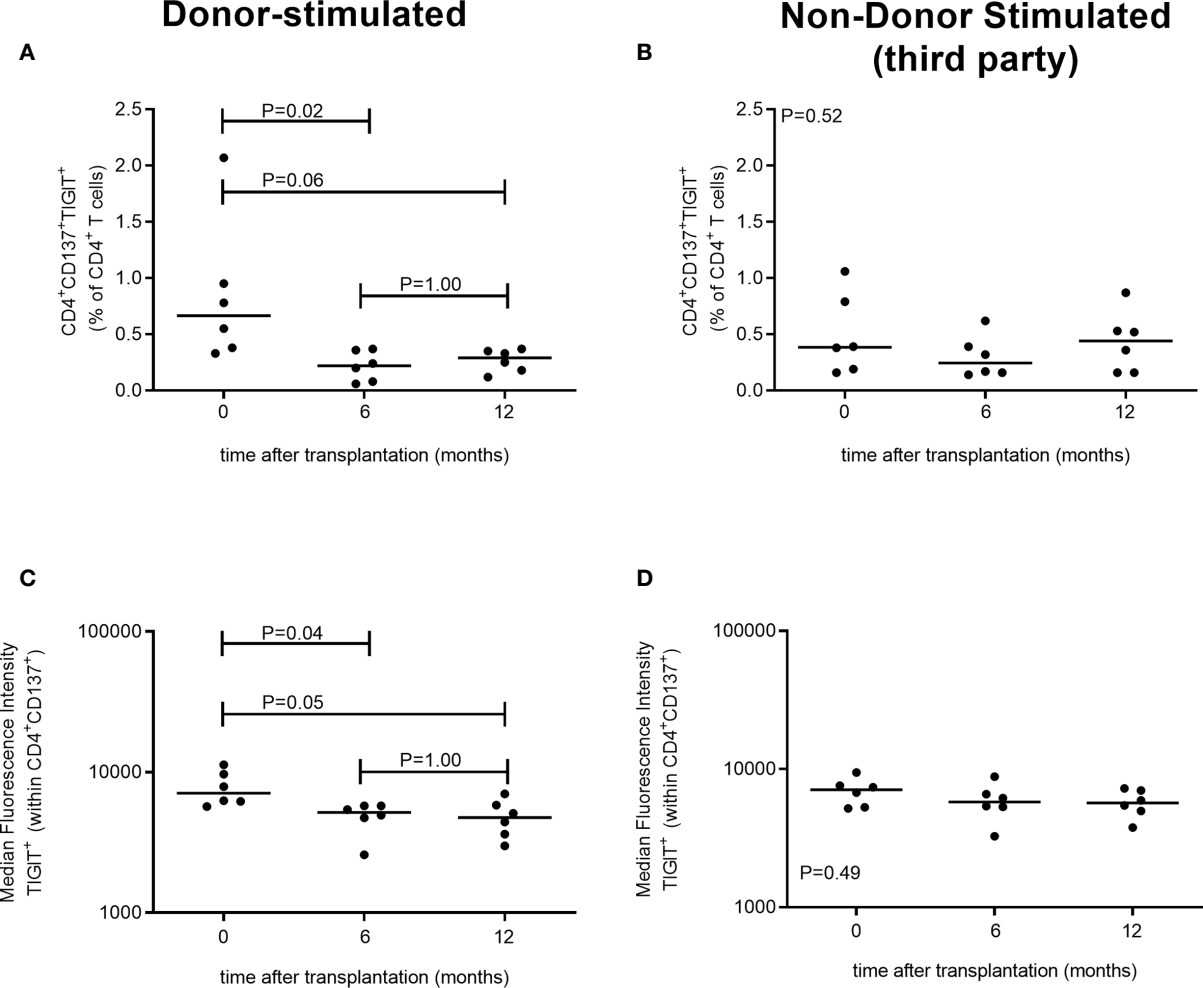
TIGIT-expressing donor-reactive CD137+ CD4+ T cells significantly decline within first 6 months following transplantation. The two panels on the left **(A**, **B)** represent PBMCs stimulated with donor-antigen while those on the left **(C**, **D)** represent PBMCs stimulated with non-donor (third party) antigen. In the top panels the percentage of CD4+ T cells co-expressing CD137 and TIGIT is illustrated prior to transplantation (timepoint 0) and at 6 and at 12 months following transplantation for 6 kidney transplant recipients per timepoint. In the bottom panels the MFI of TIGIT-expressing cells within the CD4+CD137+ T cell population is depicted.

In a separate cohort of kidney transplant recipients, we also have evaluated TIGIT expression following stimulation with donor-antigen at earlier time points, i.e. within the first year, after transplantation. The proportion of CD4+ T cells which are donor-reactive (CD137+) and express TIGIT significantly declines within the first 6 months after transplantation (P = 0.02) (**Figure 3**). After this initial decrease in the first 6 months we do not observe a further decline at a year post kidney transplantation (P=1.00) (**Figure 3A**). These observations are reflected within the MFI of TIGIT within the CD137+CD4+ population which also declines significantly from prior to month 6 after transplantation (P = 0.04) but does not decline further from month 6 to a year after transplantation (P = 1.00) (**Figure 3B**) Importantly, no significant changes in CD4+CD147+TIGIT+ percentages or TIGIT+ MFI were observed after third party stimulation indicating this decline is unique for donor-reactive T cells (**Figures 3C, D**). Similar to our observations 3-5 years post transplantation, the proportion of CD8+ T cells expressing CD137+ and TIGIT+ did not change within the first year post transplantation (**Supplementary Figure 3**).

### TIGIT-Expressing Donor-Reactive CD137high CD4+ T Cells Are Predominantly of the Memory Phenotype and Poly-Functional Cytokine Expressers

**Figure 4:**
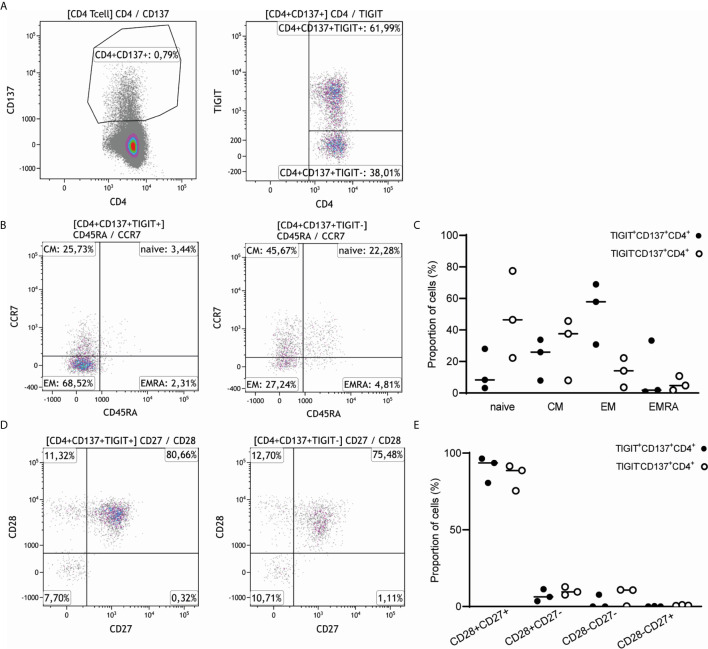
CD4+CD137+TIGIT+ T cells have a predominantly central and effector memory T cell phenotype and express both CD27 and CD28. **(A)** A representative sample of the 3 healthy individuals stimulated with alloantigen illustrates how CD4+CD137+ T cells were gated and split into a TIGIT positive and TIGIT negative fraction. **(B)** Expression of CD45RA and CCR7 was compared for both CD4+CD137+ T cells expressing TIGIT (left) and negative for TIGIT (right) of 3 healthy individuals. The percentage of gated CD4+CD137+TIGIT+ and gated CD4+CD137+TIGIT- cells are indicated within each quadrant. **(C)** Naïve, central memory (CM), effector memory (EM) or terminally differentiated effector memory (EMRA) cells are depicted as proportion of CD4+CD137+ T cells either positive (closed symbols) or negative (open symbols) for TIGIT of 3 healthy individuals. **(D)** Expression of CD27 and CD28 was illustrated for both CD4+CD137+ T cells expressing TIGIT (left) and negative for TIGIT (right). The percentage of gated CD4+CD137+TIGIT+ and gated CD4+CD137+TIGIT- cells are indicated within each quadrant. **(E)** The proportion of CD4+CD137+ T cells either positive (closed symbols) or negative (open symbols) for TIGIT and expressing CD28 and CD27 are depicted.

CD4+ T cells co-expressing CD137 and TIGIT (CD4+CD137+TIGIT+ T cells) were predominantly memory T cells, with a median (range) proportion of 52.54% (30.75-68.98) effector memory T cells (CD45RA-CCR7-) followed by 22.5% (7.96-33.75) central memory T cells (CD45RA-CCR7+) (**Figures 4A–C**). By comparison, CD4+CD137+ T cells negative for TIGIT were mainly of the naïve (48.74%, 22.26-77.49) and central memory phenotype (37.64%, 8.06-45.7) with a smaller proportion of effector memory T cells (13.33%, 3.65-22.23) (**Figures 4A–C**). The CD4+CD137+TIGIT+ T cells had a relatively higher percentage of CD28+CD27+ T cells (90.25%, 80.65-96.46) and a lower percentage of CD28-CD27- T cells (2.57%, 0-7.72) compared to TIGIT negative cells (**Figures 4D, E**).

**Figure 5:**
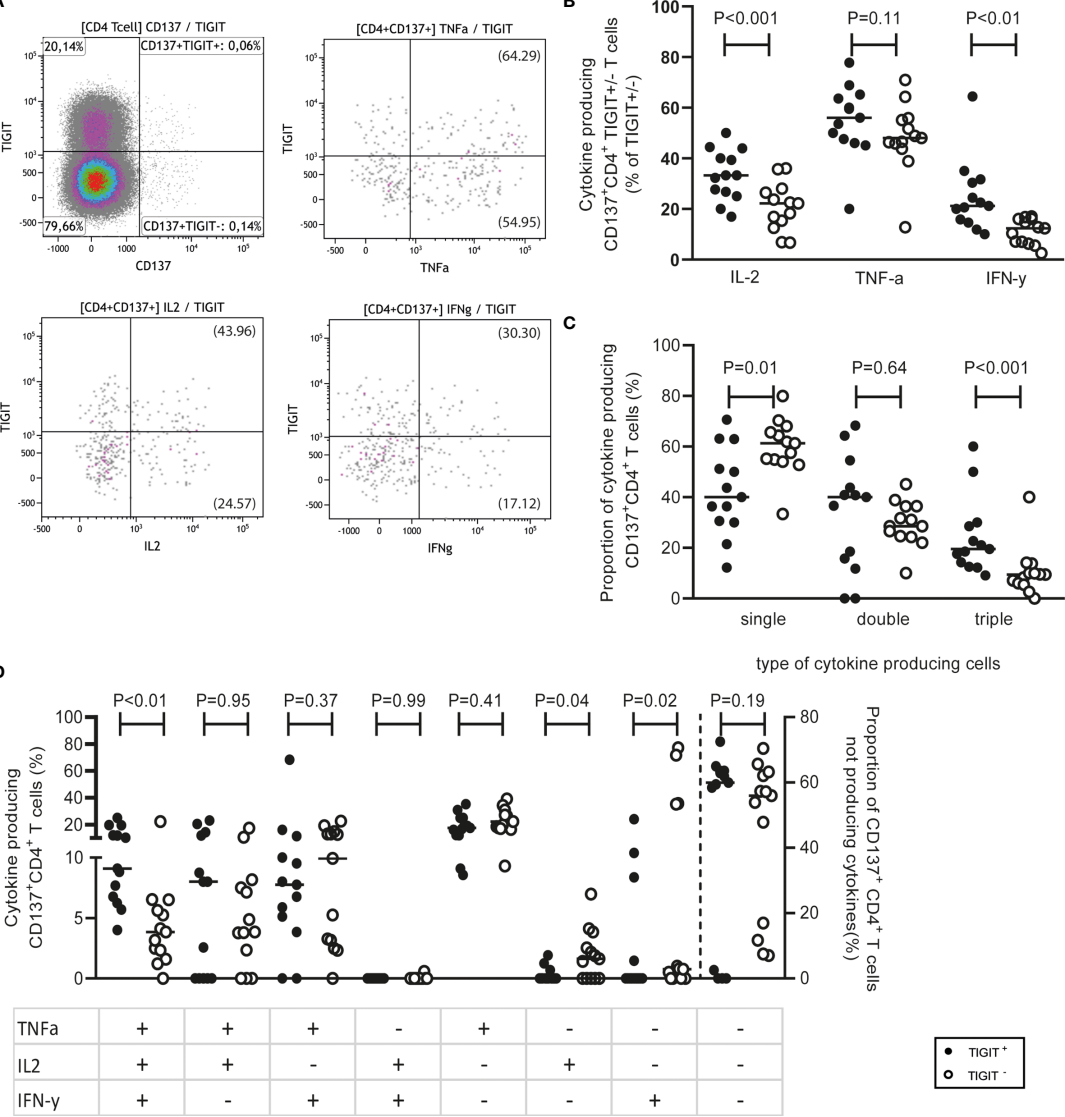
CD4+CD137+ TIGIT+ T cells secrete cytokines in a polyfunctional manner. **(A)** PBMCs obtained from 13 responders (4 healthy individuals and 9 kidney transplant recipients pre transplantation) following stimulation with alloantigen. A representative sample illustrates how CD4+CD137+ T cells were gated then analyzed for co-expression of TIGIT and the cytokines, TNF-α, IL-2 and IFN-γ. In brackets is shown the fraction of TIGIT+/- within CD137+CD4+ T cells which express the cytokine. **(B)** The percentage of cytokine-producing CD4+CD137+ T cells either positive or negative for TIGIT which produce IL-2, TNF-α and/or IFN-γ. **(C)** The proportion of cytokine producing CD4+CD137+ T cells within the TIGIT positive or negative portions which produce one (single), two (double) or all (triple) of the cytokines, TNF-α, IL-2 and IFN-γ. **(D)** The combinations of TNF-α, IL-2 and IFN-γ cytokines produced by CD4+CD137+ T cells divided according to TIGIT expression.

In terms of functional characteristics, CD4+CD137+TIGIT+ T cells produced multiple inflammatory cytokines. Compared to CD137+CD4+ T cells not expressing TIGIT, a higher proportion of CD137+CD4+ T cells expressing TIGIT secreted IL-2 (P <0.001) and IFN-γ (P <0.01). A median (range) of 33.33% (16.93-50) TIGIT-expressing CD4+CD137+ T cells secreted IL-2 and 21.21% (10-64.42) produced IFN-γ compared to 22.22% (6.7-36.05) and 12.37% (2.56-17.04) respectively secreted by TIGIT-negative CD4+CD137+ T cells (**Figures 5A, B**). In addition, CD4+CD137+TIGIT+ T cells had a higher proportion of poly-functional T cells secreting all three cytokines (TNF-α, IFN-γ and IL-2) compared to CD4+CD137+ T cells negative for TIGIT (**Figures 5C, D**; P <0.01). This is in contrast to CD137+CD4+ T cells negative for TIGIT for which the majority of 61.26% (33.34-79.97) produced only one cytokine (**Figures 5C, D**).

## Discussion

This study describes the novel finding that donor-reactive CD4+ T cells expressing TIGIT are specifically decreased several years after KT. The expression of co-inhibitory receptors such as TIGIT was hypothesized to mark exhaustion of donor-reactive T cells after continuous exposure of the recipient’s immune system to the donor kidney. Such a process could offer an explanation for donor-specific hyporesponsiveness, which develops in the years after KT and allows for lowering of immunosuppressive drugs. Instead of the expected increase of T cells expressing exhaustion markers, unbiased analysis of the multi-parameter immunophenotype data demonstrated that specifically clusters containing CD137-expressing T cells (a marker for antigen-specific, i.e. donor-reactive T cells) with a relative high TIGIT expression were decreased 3-5 years after transplantation. Of note, all these clusters were CD4+ T cells. This decrease post KT was confirmed using traditional flow cytometric analysis and was most pronounced in the elderly recipients. This is in line with the clinical observation of a decreased age-associated long-term risk for acute rejection (19).

TIGIT is a co-inhibitory receptor expressed on natural killer (NK) cells and activated T cells, including memory, follicular T helper and regulatory T cell subsets (20, 21). The expression of TIGIT functions to inhibit further activation, proliferation or maturation of activated T cells through multiple direct and indirect mechanisms. These mechanisms include downregulation of T-cell receptor activation pathways and engagement with ligands of the Nectin/Nectin-like (Necl) family expressed on antigen-presenting cells (APCs) which leads to tolerogenic APCs that secrete anti-inflammatory cytokines such as interleukein (IL)-10 (22). Binding of TIGIT to Necl family ligands also prevents the binding of competing co-stimulatory receptors such CD226 (DNAM-1) which functions to indirectly inhibit T cell activation (21, 23).

TIGIT expression on T cells has been demonstrated on dysfunctional or exhausted T cells in chronic diseases including viral immunity and cancer. In these diseases, antigen-reactive CD8+ T cells with high co-expression of TIGIT+, PD-1+ and TIM-3+ were termed ‘exhausted’ due to their loss of effector function (24, 25). Combinatorial blockade of these receptors was shown to benefit disease outcome by improving effector CD8+ T cell and natural killer cell function as well as decreasing Treg-mediated suppression (22).

Blocking of these co-inhibitory molecules by monoclonal antibodies directed against for instance PD-1 and CTLA-4 (known as immune checkpoint inhibitor therapy) has evolved in recent years as a novel and sometimes very effective immunotherapy for cancers like melanoma or lung carcinoma. The checkpoint inhibitors allow the tumor-reactive T cells to exert their immune function like cytotoxicity but also increase the risk for auto-immune diseases and in the case of solid organ transplantation, acute cellular rejection in about 20-30% of cases (26, 27). This indicates that co-inhibitory molecules on alloreactive T cells are contributing, in combination with immune-suppressive drugs, to the containment of their activity. These unwanted side effects of check-point inhibitor therapy illustrate that although accumulation of co-inhibitory molecules on T cells is associated with senescence or exhaustion, they are also present and functional on T cells that can be active once the inhibitory signal is blocked.

To our knowledge, only one other study tested the role of T-cell exhaustion in the context of KT (10). Although this study found an increase in exhausted CD4+ and CD8+ T cells 6-months post transplantation, this trend was only shown to be significant in KT recipients who received lymphocyte-depleting induction therapy with anti-thymocyte globulin (ATG). Therefore, it appears that in the case of KT recipients who receive non-depleting induction therapy (as used in the present study), the expression of commonly used markers to identify exhaustion of T cells remains unchanged in the years after transplantation and the findings are not in support of a concept of exhaustion-driven donor-specific T cell hyporesponsiveness. Interestingly, our data show that the frequency of TIGIT expressing donor-reactive CD4+ T cells already decreases significantly within the first 6 months after transplantation. The alloreactivity against non-donor HLA remains unchanged, indicating a donor-specific phenomenon and not a general effect of immunosuppressive medication.

The observation that CD4+ T cells expressing TIGIT decrease after KT is in line with publications in different areas of immunology studying conditions of chronic immunostimulation. For instance, a reduction in CD4+ T cells expressing TIGIT has also been observed in rheumatoid arthritis and psoriasis (28, 29). This suggests that chronic immune stimulation causes completely different changes in the CD4+ T cell compartment as opposed to the CD8+ T cell compartment. This has very recently also been documented for hepatitis C virus (HCV) infection. Individuals chronically infected with HCV showed an expansion of exhausted CD8 HCV-reactive T cells but lose their HCV-reactive CD4+ T cells over time, while individuals that clear the infection do not lose their HCV-reactive CD4+ T cells (30). Also, clearance of a chronic HCV infection, which may occur during pregnancy, was associated with the re-appearance of HCV-reactive CD4+ T cells (30). Of note, the HCV-reactive CD4+ T cells expressed the exhaustion marker PD-1, which was interpreted as a sign of activation and differentiation rather than exhaustion (30). In the same way, our study demonstrated that TIGIT expression on donor-reactive CD4 T cells can be interpreted as a marker for a polyfunctional memory CD4+ T subset which may pose a threat to the transplanted graft. Antigen-specific memory CD4+ T cells have been shown in animal models of transplantation to exacerbate allograft rejection by directing the effector functions of not only fellow CD4+ T cells, but also CD8+ cytotoxic T cells and alloantibody-producing B cells (31–34). A murine model by Uehara 2018 et al. concluded that alloreactive CD4+ T cells are indispensable for cytotoxic CD8+ T cell-mediated acute rejection (35).

In conclusion, our unbiased multi-parameter clustering approach identified a significant decrease in donor-reactive CD4+ T cells highly expressing TIGIT long after kidney transplantation. The reduction in this donor-reactive T cell population in stable kidney transplantation recipients is a novel finding and may contribute to the development of DSH, meriting further confirmatory investigations in larger cohorts. Prospective clinical studies could help determine whether a low frequency of donor-reactive TIGIT-expressing CD4+ T cells could guide lowering of immunosuppressive drugs.

## Data Availability Statement

The original contributions presented in the study are included in the article/**Supplementary Material**. Further inquiries can be directed to the corresponding author.

## Ethics Statement

The studies involving human participants were reviewed and approved by METC Erasmus medical center. The patients/participants provided their written informed consent to participate in this study.

## Author Contributions

NL and MB conceptualized the research goals and aims and acquired funding for the project. NL and MK designed and conceived all antibody panels for experiments and set up the FACS for measurement. NL, MK, and AL performed all experiments and analyzed FACS images. AL analyzed the FlowSOM data, wrote and edited the manuscript. MB provided helpful discussion on experiments and data analysis. NL, MB, and AL edited and all authors commented on the final manuscript. All authors contributed to the article and approved the submitted version.

## Funding

This study was financially supported by the Dutch Kidney Foundation under the GANDALF study (project number: 18PhD08).

## Conflict of Interest

The authors declare that the research was conducted in the absence of any commercial or financial relationships that could be construed as a potential conflict of interest.
