# Supplementary material for: Expression of Senescence Marker TIGIT Identifies Polyfunctional Donor-Reactive CD4+ T Cells Preferentially Lost After Kidney Transplantation
Source: Front Immunol. 2021 Apr 30;12:656846. doi: 10.3389/fimmu.2021.656846 (PMC8119878; doi:10.3389/fimmu.2021.656846)
Supplement: Supplementary file 1 [file DataSheet_1.docx]

Supplementary Material

# Supplementary Tables

## Supplementary Table 1: Antibodies

| **Marker** | **Fluorochrome** | **Titer (µL)*** | **Company** | **Clone** | **Catalog #** | **RRID** |
| --- | --- | --- | --- | --- | --- | --- |
| CD137 | APC | 5 | BD | 4B4-1 | 550890 | AB_398477 |
| CD3 | BV510 | 1 | BD | HIT3a | 564713 | AB_2738909 |
| CD4 | PerCPCy5.5 | 0.5 | BD | RPA-T4 | 560650 | AB_1727476 |
| CD8 | APC-R700 | 1 | BD | RPA-T8 | 565165 | AB_2744457 |
| CD14 | APC-H7 | 0.125 | BD | MφP9 | 560180 | AB_1645464 |
| CD19 | APC-H7 | 0.5 | BD | SJ25C1 | 560177 | AB_1645470 |
| CD56 | APC-Cy7 | 0.5 | BioLegend | HCD56 | 318332 | AB_10896424 |
| FVS780 |  | 0.25** | BD | none | 565388 |  |
| CD45RA | BV650 | 0.25 | BioLegend | HI100 | 304136 | AB_2563653 |
| CCR7 | BV421 | 0.5 | BioLegend | G042H7 | 353208 | AB_11203894 |
| CD27 | BV605 | 2.5 | BD | L128 | 562655 | AB_2744351 |
| CD28 | BV785 | 1 | BioLegend | CD28.2 | 302950 | AB_2632607 |
| CD160 | PE-Cy7 | 2.5 | BioLegend | BY55 | 341212 | AB_2562876 |
| LAG3 | BV605 | 2.5 | BD | T47-530 | 745160 | AB_2742761 |
| PD1 | BV421 | 1 | BD | MIH4 | 564323 | AB_2738745 |
| TIM3 | BV786 | 0.25 | BD | 7D3 | 742857 | AB_2741100 |
| TIGIT | PE | 0.5 | BioLegend | A15153G | 372704 | AB_2632730 |
| CD244 | FITC | 10 | BD | feb-69 | 550815 | AB_393900 |
| CTLA4 | PE-Dazzle594 | 0.25 | BioLegend | BNI3 | 369616 | AB_2632878 |
| IL-2 | BV421 | 0.5 | BD | MQ-17H12 | 564164 | AB_2738635 |
| IFN-γ | BV711 | 0.5 | BD | 4SB3 | 502540 | AB_2563506 |
| TNF-α | BV605 | 1 | BD | Mab11 | 502936 | AB_2563884 |

* µL MoAb/50 µL cell suspension volume

**µL MoAb/ 1 mL cell suspension of max 10 million cells

## Supplementary Table 2: Differential Abundance of N=9 elderly pre vs post KT T cells stimulated with 3^rd^ party antigen. TopDA clusters with a p adjusted value below 0.05.

| **cluster_id** | **p_val** | **p_adj** | **logFC** |
| --- | --- | --- | --- |
| 599 | 3.5E-05 | 0.01 | -0.86 |
| 624 | 3.6E-05 | 0.01 | -0.86 |
| 450 | 1.1E-04 | 0.02 | -0.85 |

## Supplementary Table 3: Differential Abundance of N=8 young pre vs post KT T cells stimulated with 3^rd^ party antigen. The 10 clusters with the lowest p adjusted value (NB: none are below 0.05).

| **cluster_id** | **p_val** | **p_adj** |
| --- | --- | --- |
| 5 | 0.01 | 0.222 |
| 8 | 0.01 | 0.222 |
| 30 | 0.01 | 0.222 |
| 37 | 0.005 | 0.222 |
| 57 | 0.008 | 0.222 |
| 59 | 0.008 | 0.222 |
| 63 | 0.006 | 0.222 |
| 72 | 0.004 | 0.222 |
| 130 | 0.003 | 0.222 |
| 137 | 0.01 | 0.222 |

#
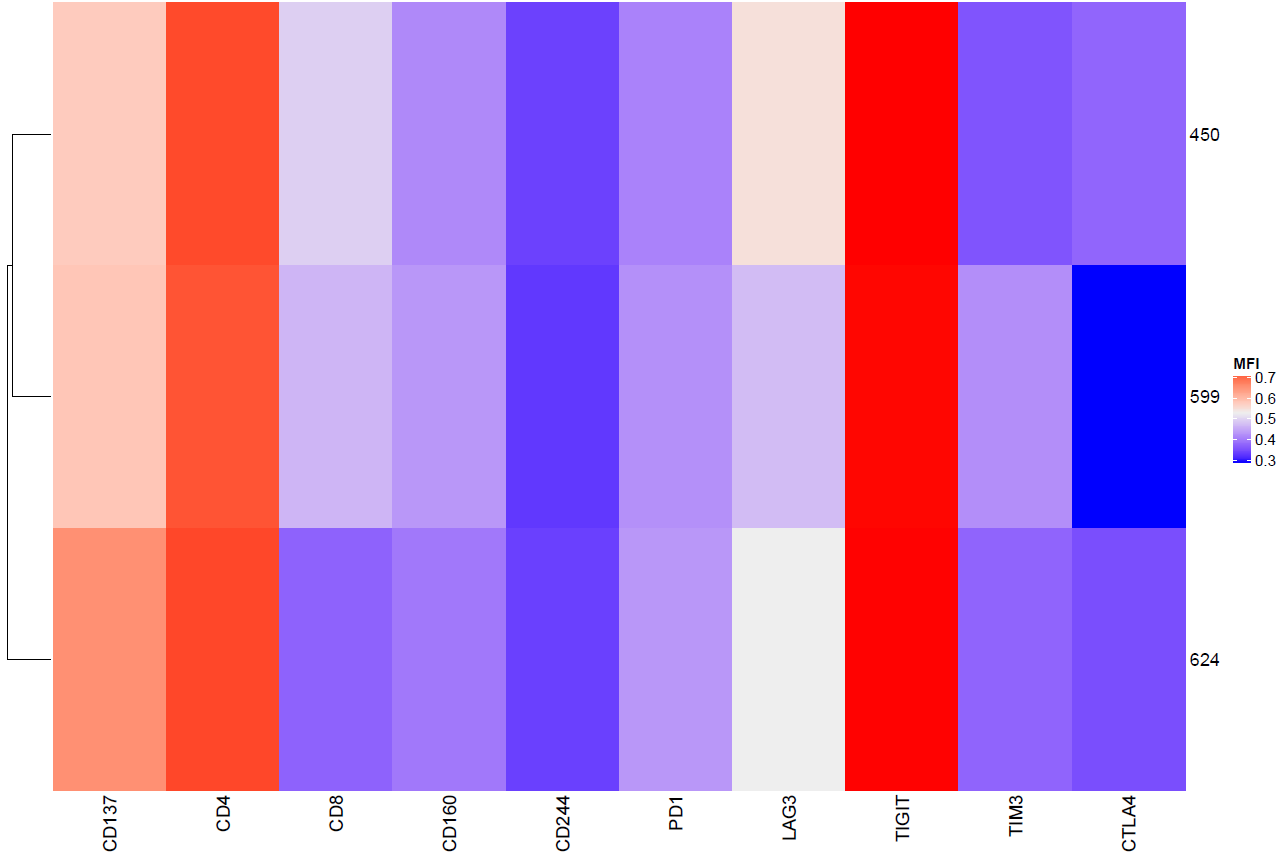
Supplementary Figures

## Supplementary Figure 1. Heatmap of topDA clusters (p_adj < 0.05) resulting from diffcyt analysis of pre vs post of N=9 elderly KT with 3rd party antigen FlowSOM clustering


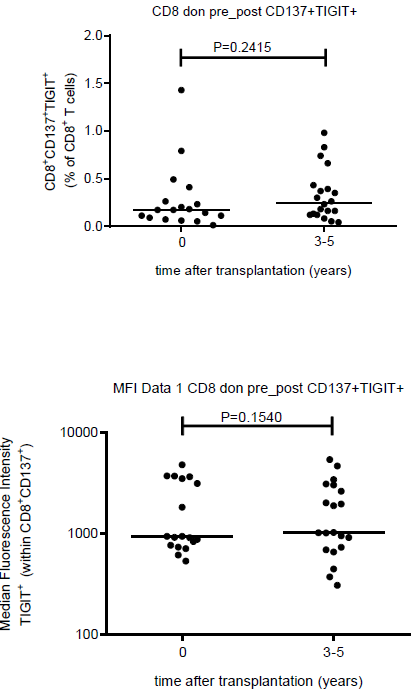


**Supplementary Figure 2**. Percentage and MFI of TIGIT-expressing donor-specific CD8+ T cells remains unchanged 3-5 years post transplantation. In the top panel, percentage of CD8+ T cells with expression of CD137 and TIGIT is illustrated prior to transplantation (timepoint 0) and 3-5 years post transplantation for 17 kidney transplant recipients. In the bottom panels these same samples are represented by the MFI of TIGIT-expressing cells within the CD8+CD137+ T cell population.

**Donor-stimulated**


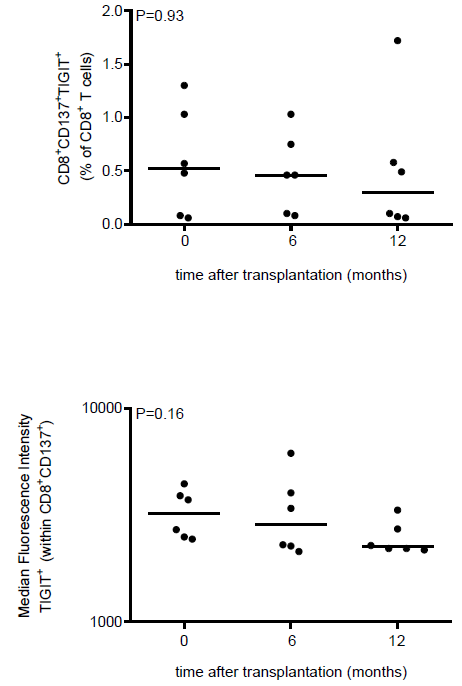


**Third-party stimulated**


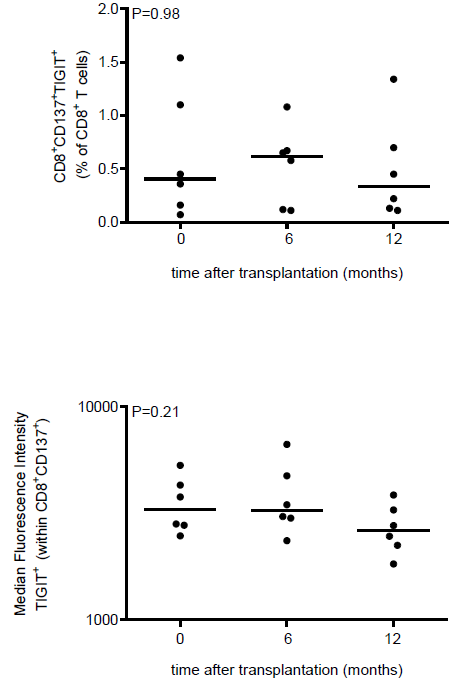


**Supplementary Figure 3**. Percentage and MFI of TIGIT-expressing donor-specific CD137+ CD8+ T cells remain unchanged within first 12 months post transplantation. The two panels on the left represent PBMCs stimulated with donor-antigen while those on the right represent PBMCs stimulated with third-party antigen. In the top panels the percentage of CD8+ T cells co-expressing CD137 and TIGIT is illustrated prior to transplantation (timepoint 0) and at 6 and at 12 months post transplantation for 6 kidney transplant recipients per timepoint. In the bottom panels the MFI of TIGIT-expressing cells within the CD8+CD137+ T cell population is depicted.
